# Neurological Symptoms and Comorbidities in Patients with Ankylosing Spondylitis

**DOI:** 10.3390/jcm15093325

**Published:** 2026-04-27

**Authors:** Ece Köse, Mustafa Serhan Sevim

**Affiliations:** 1 Neurology Clinic, Bucak State Hospital, 15300 Burdur, Türkiye; 2 Department of Neurology, Mersin University Faculty of Medicine, 33110 Mersin, Türkiye; serhansevim33@gmail.com

**Keywords:** ankylosing spondylitis, autonomic dysfunction, BASDAI, insomnia, neuropathic pain

## Abstract

**Background/Objective**: This study aimed to determine the prevalence of neurological symptoms and findings in patients with Ankylosing Spondylitis (AS) and to evaluate their relationship with disease activity. **Methods**: A total of 86 patients diagnosed with AS underwent a structured neurological examination including assessment of mental status, cranial nerves, motor system, deep tendon reflexes, pathological reflexes, and cerebellar/extrapyramidal functions. Sensory deficits and motor weakness were recorded. Orthostatic hypotension was evaluated as a clinical marker of autonomic involvement. Insomnia symptoms and neuropathic pain features were assessed clinically. Disease activity was quantified using the Bath Ankylosing Spondylitis Disease Activity Index (BASDAI). Associations between neurological findings, BASDAI scores, and inflammatory parameters were analyzed. **Results**: Motor weakness was observed in 5% of patients. Sensory deficits were present in 31% and orthostatic hypotension in 23% of patients. Insomnia symptoms were reported by 51% and neuropathic pain features by 53% of participants. A highly significant association was found between insomnia and neuropathic pain (*p* < 0.001). BASDAI scores were significantly higher in patients with insomnia, orthostatic hypotension, and sensory deficits (*p* = 0.004, *p* = 0.014, and *p* < 0.001, respectively). No significant association was observed between Anti-Tumor Necrosis Factor therapy and sensory deficits, and no significant correlation was demonstrated between neurological findings and C-reactive protein/Erythrocyte Sedimentation Rate values (*p* > 0.05). **Conclusions**: Neurological symptoms are common in AS and are associated with higher disease activity, without parallel changes in inflammatory markers such as CRP and ESR. Systematic evaluation of these symptoms may facilitate earlier identification of subgroups with a higher disease burden and inform individualized follow-up and management strategies.

## 1. Introduction

Ankylosing spondylitis (AS) is a chronic inflammatory disease that primarily affects the axial skeleton and is the prototype of the spondyloarthropathies group [1,2]. The disease typically begins in young adulthood and is characterized by inflammatory back pain, morning stiffness, and progressive spinal mobility restriction. However, AS is not limited to the musculoskeletal system; it is a systemic condition that can manifest with various extra-articular features including uveitis, cardiovascular disease, and neurological involvement [3].

Neurological involvement in AS has traditionally been described in the context of mechanical and structural complications such as vertebral fractures, subluxations, or cauda equina syndrome [4,5]. However, recent studies have demonstrated that nervous system involvement is not confined to mechanical processes alone, and that chronic systemic inflammation may have direct effects on both the central and peripheral nervous systems. This involvement can manifest clinically across a broad spectrum including neuropathic pain, sensory deficits, autonomic dysfunction, and sleep disturbances [6]. Autonomic nervous system dysfunction, in particular, has been associated with impaired cardiovascular autonomic regulation in patients with ankylosing spondylitis, potentially leading to clinical manifestations such as orthostatic hypotension (OH) and arrhythmias [7].

In rheumatic diseases, in addition to pain and functional limitation, the modulatory effects of pro-inflammatory cytokines on sleep architecture and pain perception have attracted considerable attention. The presence of impaired sleep quality and a neuropathic pain component in patients with AS has been reported to be closely associated with disease activity and quality of life [8,9]. Chronic systemic inflammation may also affect the central nervous system, and population-based studies suggest a possible increased risk of neurodegenerative disorders in patients with AS [10].

Neurological symptoms are often nonspecific and may be overlooked during routine rheumatology consultations. However, early detection of these findings is critical for improving patients’ functional status and enhancing their quality of life. Despite reports in the literature highlighting the presence of neurological complaints in AS, data combining practical assessment methods such as clinical examination and orthostatic blood pressure measurements to establish their relationship with disease activity remain limited.

Therefore, this study aimed to determine the prevalence of key neurological symptoms and to evaluate their association with disease activity (BASDAI) in patients with AS.

## 2. Materials and Methods

### 2.1. Study Design and Ethical Approval

This cross-sectional study was planned by the Department of Neurology, Mersin University Faculty of Medicine, and conducted in patients with AS who were being followed at the Rheumatology Outpatient Clinic and fulfilled the Modified New York (1984) criteria [11]. The study protocol was approved by the Mersin University Clinical Research Ethics Committee (Date: 1 April 2020; Approval No: KAEK/2020/272). Written informed consent was obtained from all participants.

### 2.2. Participants and Eligibility Criteria

A total of 86 patients with AS, aged 18–70 years, who attended routine follow-up visits at the Rheumatology Outpatient Clinic between 15 April 2020 and 15 June 2020 were included. Exclusion criteria were pregnancy; anemia; hyperthyroidism or hypothyroidism; a diagnosis of diabetes mellitus; use of additional medications or herbal products other than those for AS; the presence of a known primary neurological or neuromuscular disorder; and refusal to participate. Participants who consented initially but were later found to have an additional comorbid condition were excluded from the study.

### 2.3. Demographic and Clinical Assessments

Demographic characteristics (age and sex) were recorded. Height and weight were measured, and body mass index (BMI) was calculated. The date of AS diagnosis, current treatments used within the last 6 months, and smoking status were documented. Smoking was defined as smoking at least one cigarette per day within the past 6 months; pack-years were calculated based on the number of packs smoked per day multiplied by the duration of smoking in years. Disease activity was assessed by a rheumatologist using the BASDAI [12]. BASDAI consists of six questions assessing fatigue, spinal pain, peripheral joint pain/swelling, localized tenderness, and morning stiffness, and yields a total score ranging from 0 to 10.

### 2.4. Neurological Evaluation Protocol

All participants underwent detailed history-taking focused on neurological symptoms and a comprehensive neurological examination. The examination included assessment of ocular motility (gaze limitation), muscle strength, sensory function, deep tendon reflexes and pathological reflexes, cerebellar and extrapyramidal system examinations, as well as gait and balance assessment. For evaluation of autonomic nervous system involvement, symptoms such as palpitations, dizziness, blurred vision/blackouts, hyperhidrosis, and anhidrosis were queried. Orthostatic blood pressure and heart rate measurements were obtained by the investigator according to a standardized protocol. OH was defined as a decrease of ≥20 mmHg in systolic blood pressure or ≥10 mmHg in diastolic blood pressure upon standing [13]. Insomnia symptoms and neuropathic pain features were assessed based on patient-reported complaints during clinical evaluation, without the use of standardized questionnaires.

### 2.5. Statistical Analysis

Data analysis was performed using SPSS Statistics for Windows, version 24.0 (IBM Corp., Armonk, NY, USA). The normality of continuous variables was assessed using visual methods (histograms, probability plots) and analytical tests (Kolmogorov–Smirnov/Shapiro–Wilk tests). Descriptive statistics were expressed as mean ± standard deviation or median and interquartile range (IQR) for continuous variables, and as frequency and percentage for categorical variables. Student’s *t*-test was used to compare differences between two independent groups with normal distribution, while the Mann–Whitney U test was applied for nonnormally distributed variables. Chi-square test was used for the analysis of categorical variables; Fisher’s exact test was preferred when expected cell counts were low. Associations between categorical variables were evaluated using chi-square or Fisher’s exact tests as appropriate. Statistical significance was set at *p* < 0.05 for all analyses.

## 3. Results

A total of 86 patients with AS were included in the study. The mean age of the patients was 42.3 ± 9.4 years, and the sex distribution was 64% male and 36% female. The mean body mass index (BMI) was 27.6 kg/m^2^ and 31% of patients were current smokers. The distribution of disease duration was found to be <1 year in 26%, 1–5 years in 20%, and >5 years in 54% of the cohort. Mean inflammatory markers were measured, with C-reactive protein (CRP) at 10.1 ± 6.3 mg/L and erythrocyte sedimentation rate (ESR) at 13.7 ± 8.2 mm/hour. The mean disease activity measured by BASDAI was 4.31 ± 1.52. Anti-TNF therapy was administered to 40.7% of the patients (Table 1).

Neurological examination revealed no marked deficits in mental status, cranial nerves, deep tendon reflexes, pathological reflexes, or cerebellar and extrapyramidal system assessments. However, motor system evaluation demonstrated muscle strength deficit in 5% of the patients (*n* = 5). Regarding the frequency of symptoms and findings, sensory deficits were identified in 27 patients (31%) and OH in 20 patients (23%). Insomnia symptoms were reported by 44 patients (51%), and neuropathic pain features by 46 patients (53%) (Table 2).

In addition, a highly significant association was observed between insomnia and neuropathic pain (*p* < 0.001). Neuropathic pain was present in all patients with insomnia (100%), whereas it was observed in only 4.8% of those without insomnia. Neuropathic pain was also observed more frequently in female patients (64%) than in male patients (47%).

When the relationship between disease activity and neurological findings was evaluated, the mean BASDAI score was 4.82 ± 1.78 in patients with insomnia and 3.79 ± 1.43 in those without insomnia, with the difference being statistically significant (*p* = 0.004). Similarly, patients with OH had a mean BASDAI score of 5.13 ± 1.88, compared with 4.07 ± 1.56 in those without OH (*p* = 0.014). BASDAI was also significantly higher in patients with sensory deficits (5.28 ± 1.56) than in those without sensory deficits (3.88 ± 1.57) (*p* < 0.001) (Table 3).

In contrast, no significant association was found between anti-TNF therapy use and the presence of sensory deficits (*p* > 0.05). Furthermore, no statistically significant correlation was demonstrated between neurological findings and inflammatory laboratory parameters, including CRP and ESR (*p* > 0.05).

## 4. Discussion

Ankylosing spondylitis (AS) is characterized not only by axial skeletal involvement but also by a broad spectrum of extra-articular manifestations driven by systemic inflammation. Although the literature largely emphasizes spinal deformity and peripheral joint involvement, the relationship between neurological symptoms and overall disease burden and activity remains insufficiently elucidated. The present study examines the prevalence of neurological features—including sensory deficits, autonomic dysfunction, insomnia, and neuropathic pain—in patients with AS and explores their associations with clinical parameters. Our findings suggest that neurological involvement may represent a clinical pattern associated with higher disease activity.

In our cohort, the mean age of patients with AS was 42.3 ± 9.4 years, with most individuals falling within the middle-aged group. Although AS typically has its onset in the second to third decades of life [1,14,15], the relatively higher mean age observed in our study likely reflects the age at the time of outpatient follow-up rather than age at disease onset. This may be attributable to the chronic and progressive course of AS, prolonged duration of clinical follow-up, and the tendency for tertiary-care populations to include patients with older age and longer disease duration.

With respect to sex distribution, 64% of the participants were male (male-to-female ratio ≈ 1.8), consistent with the male predominance reported in the literature [1,14,15]. This pattern may be related to the higher prevalence of AS among men as well as sex-related differences in clinical spectrum and healthcare-seeking behaviors.

In the present study, 31% of patients were current smokers. Smoking in AS has been associated with worse clinical activity and greater functional impairment. For example, Kaan et al. [16] reported significantly higher BASDAI and Bath Ankylosing Spondylitis Functional Index (BASFI) scores among smokers with AS. Similarly, Sakellariou et al. demonstrated that active smoking is independently associated with higher BASDAI scores and that increasing pack-years correlate with progression of radiographic damage as assessed by the modified Stoke Ankylosing Spondylitis Spine Score (mSASSS) [16,17]. Given that higher BASDAI scores were also associated with neurological symptoms in our cohort, factors such as smoking that exacerbate disease activity may indirectly contribute to an increased risk of neurological involvement. Accordingly, the smoking rate observed in our sample was considered a potentially important confounder influencing disease burden in our analyses.

The mean BMI in our cohort was 27.6 kg/m^2^, indicating that patients were generally in the overweight category. The adverse impact of increased BMI on disease activity and treatment response in AS is well documented. In a study by Liew et al. [18], overweight and obese individuals exhibited significantly higher BASDAI and Ankylosing Spondylitis Disease Activity Score (ASDAS) compared with those with normal BMI. Moreover, Ottaviani et al. [19] and Vodnizza et al. [20] showed that higher BMI and fat mass are associated with reduced response to anti-TNF therapies (e.g., infliximab). Considering that approximately 41% of our patients were receiving anti-TNF therapy and the mean BASDAI score was 4.31 (moderate-to-high disease activity), the prevalence of being overweight in our cohort may plausibly represent an additional factor that negatively influences both disease activity and treatment outcomes, in line with the existing literature.

Consistent with the literature, no overt central nervous system deficits were observed in our study. Nevertheless, the presence of a small subgroup with motor deficits (5%) is noteworthy and may reflect peripheral nerve involvement or mechanical complications. In addition, significant neurological symptoms were identified, including sensory deficits (31%), OH (23%), insomnia (51%), and neuropathic pain (53%). These findings demonstrate that neurological involvement in AS may encompass a broad spectrum, including peripheral, autonomic, and pain-processing mechanisms [4].

The observed rate of sensory deficits (31%) supports the possibility of peripheral nervous system involvement in AS. Gündüz et al. reported electrophysiological evidence of peripheral involvement in 18.8% of asymptomatic patients [21]. The higher clinical prevalence in our study may be related to differences in examination methods and the potential coexistence of radiculopathy or entrapment neuropathies.

The prevalence of OH (23%) in our cohort represents a clinical manifestation of autonomic dysfunction. Toussirot et al. [22] identified impaired baroreflex responses and reduced parasympathetic activity in AS, suggesting a potential association with elevated systemic inflammation. Syngle et al. [23] further reported that cardiovascular autonomic dysfunction may improve with treatment. These findings underscore the importance of monitoring autonomic involvement in relation to quality of life and cardiovascular risk.

The prevalence of insomnia in our study (51%) is consistent with reported rates ranging from 35% to 90% in the literature [24,25]. The ASLEEP study demonstrated a strong association between poor disease control (BASDAI ≥ 4) and insomnia severity [26]. Thus, sleep disturbance appears to interact bidirectionally with pain and overall disease burden.

The presence of neuropathic pain features in 53% of our patients indicate that pain in AS is not solely inflammatory in origin. Wu et al. [27] reported neuropathic components in AS using the painDETECT questionnaire. In a meta-analysis, Kim et al. [9] found that patients with neuropathic pain had higher BASDAI and BASFI scores, although inflammatory markers (ESR/CRP) did not consistently differ. The higher frequency of these features among women in our cohort (64% vs. 47%) aligns with sex-based differences in pain sensitivity reported in the literature.

Overall, neurological symptoms are common in AS, and their presence may indicate a more severe clinical phenotype independent of inflammatory parameters.

Our data indicate a statistically significant and consistent association between disease activity and selected neurological symptoms/findings. BASDAI was significantly higher in patients with insomnia than in those without insomnia (*p* = 0.004). Likewise, BASDAI was increased in patients with OH (*p* = 0.014) and was markedly higher among those with sensory deficits (*p* < 0.001). Taken together, these findings suggest that certain neurological symptoms in AS may reflect a higher overall disease burden. The available evidence further supports a close relationship between inadequate disease control and sleep disturbances in AS. In the ASLEEP study, insomnia severity was reported to be substantially higher among patients with BASDAI ≥ 4, which is consistent with our finding of higher BASDAI in patients with insomnia [26]. Moreover, large-scale studies have emphasized that impaired sleep quality is associated with pain and BASDAI, and that psychological factors—particularly anxiety—may contribute to this relationship [28].

With regard to pain phenotype and potential mechanisms underlying neurological symptoms, prior studies have suggested that neuropathic-like features may be prominent in a distinct subgroup of patients with AS and may be accompanied by less favorable clinical indicators, including higher BASDAI. painDETECT-based investigations indicate that neuropathic-like pain features can be associated with higher BASDAI and that certain clinical distinctions may be observed independently of objective inflammatory markers [9,29]. In this context, the absence of a significant correlation between CRP/ESR and neurological findings in our study suggests that some of these symptoms may be influenced by non-inflammatory mechanisms, including central sensitization or autonomic dysfunction.

Accordingly, the presence of insomnia, OH, and sensory deficits was significantly associated with higher BASDAI. From a clinical standpoint, incorporating a structured and systematic assessment of these symptoms into routine follow-up may facilitate earlier identification of patient subgroups with a higher disease burden.

This study has several limitations. Its cross-sectional design prevents causal inferences regarding the relationship between neurological symptoms and disease activity AS. The single-center setting and relatively small sample size may limit generalizability. Neurological involvement was mainly assessed through clinical evaluation and orthostatic measurements, while objective tests such as electrophysiological or autonomic function studies were not routinely performed, possibly underestimating subclinical findings. In addition, insomnia and neuropathic pain were based on symptom reporting rather than standardized questionnaires, and residual confounding factors may have influenced the results. Further longitudinal multicenter studies with objective assessments are warranted.

## 5. Conclusions

This study provides a comprehensive perspective on the frequency and clinical relevance of neurological symptoms in ankylosing spondylitis. Our findings demonstrate that insomnia, OH, and sensory deficits are significantly and consistently associated with higher BASDAI. Notably, these associations were observed without significant association with inflammatory markers, suggesting that disease burden in AS is not confined to peripheral joint and spinal involvement but may indicate a broader clinical profile potentially involving central and autonomic mechanisms. The presence of modifiable factors such as cigarette smoking and increased BMI may further amplify this burden. Therefore, the systematic assessment of neurological symptoms in patients with AS may support more comprehensive clinical assessment and help identify patients with higher disease burden.

## Figures and Tables

**Table 1:** Demographic and clinical characteristics of the study population.

Characteristic	Total *n* (%), *n* = 86
Age (years, mean ± SD)	42.3 ± 9.4
Sex (male/female)	55 (64.0)/31 (36.0)
Smoking status (yes/no)	27 (31.4)/59 (68.6)
BMI (kg/m^2^, mean)	27.6 ± 4.1
**Disease duration**	
*<1 year*	22 (26)
*1–5 years*	17 (20)
*>5 years*	47 (54)
CRP (mg/L, mean)	10.1 ± 6.3
ESR (mm/h, mean)	13.7 ± 8.2
BASDAI score (mean)	4.31 ± 1.52
Anti-TNF therapy	35 (40.7)

**Abbreviations:** SD, standard deviation; BMI, body mass index; CRP, C-reactive protein; ESR, erythrocyte sedimentation rate; BASDAI, Bath Ankylosing Spondylitis Disease Activity Index; anti-TNF, anti-tumor necrosis factor.

**Table 2:** Frequency and distribution of neurological manifestations in patients with ankylosing spondylitis (*n* = 86).

Manifestation	Present, *n* (%)	Absent, *n* (%)
Sensory deficit	27 (31)	59 (69)
Orthostatic hypotension	20 (23)	66 (77)
Insomnia	44 (51)	42 (49)
Neuropathic pain	46 (53)	40 (47)

Note: Percentages are rounded and calculated based on *n* = 86.

**Table 3:** Comparison of BASDAI scores according to the presence of neurological symptoms and findings.

Neurological Symptom/Finding	Absent Median (IQR)	Present Median (IQR)	*p*-Value
Insomnia	3.79 (2.9–4.6)	4.9 (3.6–6.1)	***0.004***
Orthostatic hypotension	4.1 (3.0–5.2)	5.2 (4.1–6.4)	***0.014***
Sensory deficit	3.9 (2.8–5.0)	5.4 (4.2–6.3)	***<0.001***

**Abbreviations:** BASDAI, Bath Ankylosing Spondylitis Disease Activity Index. Mann–Whitney U test was used (*p* < 0.05). Note: For orthostatic hypotension, *n* = 20 was used to remain consistent with the thesis results text.

## Data Availability

The data that support the findings of this study are available from the corresponding author upon reasonable request.

## Abbreviations

The following abbreviations are used in this manuscript:

AS	Ankylosing Spondylitis
BASDAI	Bath Ankylosing Spondylitis Disease Activity Index
BASFI	Bath Ankylosing Spondylitis Functional Index
ASDAS	Ankylosing Spondylitis Disease Activity Score
BMI	Body Mass Index
CRP	C-Reactive Protein
ESR	Erythrocyte Sedimentation Rate
OH	Orthostatic Hypotension
TNF	Tumor Necrosis Factor
IQR	Interquartile Range
OR	Odds Ratio
CI	Confidence Interval
SD	Standard Deviation

## References

[1] Ebrahimiadib N., Berijani S., Ghahari M., Pahlaviani F.G. (2021). Ankylosing spondylitis. J. Ophthalmic Vis. Res..

[2] Wei Y., Zhang S., Shao F., Sun Y. (2025). Ankylosing spondylitis: From pathogenesis to therapy. Int. Immunopharmacol..

[3] Ouardi N.E., Djossou J.H., Taoubane L., Ghassem M.A., Toufik H., Majjad A., Sadni S., Hmamouchi I., Abouqal R., Bahiri R. (2022). Extra-articular manifestations in patients with ankylosing spondylitis: Baseline characteristics from the RBSMR study. Mediterr. J. Rheumatol..

[4] Tyrrell P.N., Davies A.M., Evans N. (1994). Neurological disturbances in ankylosing spondylitis. Ann. Rheum. Dis..

[5] Tu P.H., Liu Z.H., Yeap M.C., Liu Y.T., Li Y.C., Huang Y.C., Lin T.M., Chen C.C. (2022). Spinal cord injury and spinal fracture in patients with ankylosing spondylitis. BMC Emerg. Med..

[6] Bengtsson K., Forsblad-d’Elia H., Deminger A., Klingberg E., Dehlin M., Exarchou S., Lindström U., Askling J., Jacobsson L.T.H. (2021). Incidence of extra-articular manifestations in ankylosing spondylitis, psoriatic arthritis and undifferentiated spondyloarthritis: Results from a national register-based cohort study. Rheumatology.

[7] Candemir M., Candemir B., Ertürk A. (2020). Evaluation of cardiac autonomic nervous system in patients with ankylosing spondylitis using 12-lead electrocardiography and Holter monitoring. Clin. Rheumatol..

[8] Salari N., Sadeghi N., Hosseinian-Far A., Hasheminezhad R., Khazaie H., Shohaimi S., Mohammadi M. (2023). Prevalence of sleep disturbance in patients with ankylosing spondylitis: A systematic review and meta-analysis. Adv. Rheumatol..

[9] Kim T.W., Son S.M., Lee J.S. (2020). Neuropathic pain in ankylosing spondylitis: A meta-analysis. Z. Rheumatol..

[10] Luo A., Yang Q., Zhang Z., Yang Y., Li X., Deng Y., He L., Zhou M. (2025). Association between ankylosing spondylitis and neurodegenerative diseases: Systematic review and meta-analysis. Jt. Bone Spine.

[11] van der Linden S., Valkenburg H.A., Cats A. (1984). Evaluation of diagnostic criteria for ankylosing spondylitis: A proposal for modification of the New York criteria. Arthritis Rheum..

[12] Garrett S., Jenkinson T., Kennedy L.G., Whitelock H., Gaisford P., Calin A. (1994). A new approach to defining disease status in ankylosing spondylitis: The Bath Ankylosing Spondylitis Disease Activity Index. J. Rheumatol..

[13] Freeman R., Wieling W., Axelrod F.B., Benditt D.G., Benarroch E., Biaggioni I., Cheshire W.P., Chelimsky T., Cortelli P., Gibbons C.H. (2011). Consensus statement on the definition of orthostatic hypotension, neurally mediated syncope and the postural tachycardia syndrome. Clin. Auton. Res..

[14] Agrawal P., Tote S., Sapkale B. (2024). Diagnosis and treatment of ankylosing spondylitis. Cureus.

[15] Ward M.M., Deodhar A., Gensler L.S., Dubreuil M., Yu D., Khan M.A., Haroon N., Borenstein D., Wang R., Biehl A. (2019). 2019 update of the American College of Rheumatology/Spondylitis Association of America/Spondyloarthritis Research and Treatment Network recommendations for the treatment of ankylosing spondylitis and nonradiographic axial spondyloarthritis. Arthritis Rheumatol..

[16] Kaan U., Ferda O. (2005). Evaluation of clinical activity and functional impairment in smokers with ankylosing spondylitis. Rheumatol. Int..

[17] Sakellariou G.T., Anastasilakis A.D., Kenanidis E., Potoupnis M., Tsiridis E., Savvidis M., Kartalis N., Sayegh F.E. (2015). The effect of smoking on clinical and radiographic variables, and acute phase reactants in patients with ankylosing spondylitis. Rheumatol. Int..

[18] Liew J.W., Huang I.J., Louden D.N., Singh N., Gensler L.S. (2020). Association of body mass index on disease activity in axial spondyloarthritis: Systematic review and meta-analysis. RMD Open.

[19] Ottaviani S., Allanore Y., Tubach F., Forien M., Gardette A., Pasquet B., Palazzo E., Meunier M., Hayem G., Job-Deslandre C. (2012). Body mass index influences the response to infliximab in ankylosing spondylitis. Arthritis Res. Ther..

[20] Ibáñez Vodnizza S.E., Nurmohamed M.T., Visman I.M., van Denderen J.C., Lems W.F., Jaime F., van der Horst-Bruinsma I.E. (2017). Fat mass lowers the response to tumor necrosis factor-α blockers in patients with ankylosing spondylitis. J. Rheumatol..

[21] Gündüz O.H., Kiralp M.Z., Ozçakar L., Cakar E., Yildirim P., Akyuz G. (2010). Nerve conduction studies in patients with ankylosing spondylitis. J. Natl. Med. Assoc..

[22] Toussirot E., Bahjaoui-Bouhaddi M., Poncet J.C., Cappelle S., Henriet M.T., Wendling D., Regnard J. (1999). Abnormal autonomic cardiovascular control in ankylosing spondylitis. Ann. Rheum. Dis..

[23] Syngle A., Verma I., Krishan P., Garg N., Syngle V. (2015). Disease-modifying anti-rheumatic drugs improve autonomic neuropathy in arthritis: DIANA study. Clin. Rheumatol..

[24] Cai Y., Chen J., Dou J., Zhou N., Shao H., Shen X., Hong M., Chen J., Fan X., Hu Q. (2025). Relationships between emotional state, sleep disturbance and health-related quality of life in patients with axial spondyloarthritis. Clin. Rheumatol..

[25] Song B.W., Jeong H.J., Kim B.Y., Cho Y.W., Son C.N., Kim S.S., Kim S.H. (2021). Bath Ankylosing Spondylitis Disease Activity Index is associated with the quality of sleep in ankylosing spondylitis patients. J. Rheum. Dis..

[26] Tymms K., Butcher B.E., Sletten T.L., Smith T., O’Sullivan C., Littlejohn G., Sadler R., Tronnberg R., Griffiths H. (2022). Prevalence of sleep disturbance and the association between poor disease control in people with ankylosing spondylitis within the Australian clinical setting (ASLEEP study): A real-world observational study using the OPAL dataset. Clin. Rheumatol..

[27] Wu Q., Inman R.D., Davis K.D. (2013). Neuropathic pain in ankylosing spondylitis: A psychophysics and brain imaging study. Arthritis Rheum..

[28] Li Y., Zhang S., Zhu J., Du X., Huang F. (2012). Sleep disturbances are associated with increased pain, disease activity, depression, and anxiety in ankylosing spondylitis: A case-control study. Arthritis Res. Ther..

[29] Choi J.H., Lee S.H., Kim H.R., Lee K.A. (2018). Association of neuropathic-like pain characteristics with clinical and radiographic features in patients with ankylosing spondylitis. Clin. Rheumatol..

